# Antibody Neutralization of HIV-1 Crossing the Blood-Brain Barrier

**DOI:** 10.1128/mBio.02424-20

**Published:** 2020-10-20

**Authors:** Valérie Lorin, Anne Danckaert, Françoise Porrot, Olivier Schwartz, Philippe V. Afonso, Hugo Mouquet

**Affiliations:** a Laboratory of Humoral Immunology, Department of Immunology, Institut Pasteur, Paris, France; b INSERM U1222, Paris, France; c Université de Paris, Sorbonne Paris Cité, Paris, France; d UTechS Photonic BioImaging, C2RT, Institut Pasteur, Paris, France; e Virus & Immunity Unit, Department of Virology, Institut Pasteur, Paris, France; f CNRS URA3015, Paris, France; g Oncogenic Virus Epidemiology and Pathophysiology Unit, Department of Virology, Institut Pasteur, Université de Paris, Paris, France; h CNRS UMR 3569, Université de Paris, Paris, France; McMaster University

**Keywords:** antibodies, blood brain barrier, HIV-1, neutralization, transcytosis

## Abstract

HIV-1 can cross the blood-brain barrier (BBB) to penetrate the brain and infect target cells, causing neurocognitive disorders as a result of neuroinflammation and brain damage. Here, we examined whether antibodies targeting the HIV-1 envelope glycoproteins interfere with the transcytosis of virions across the human BBB endothelium. We found that although the viral envelope spike gp160 is required for optimal endothelial cell endocytosis, no anti-gp160 antibodies blocked the BBB transcytosis of HIV-1 *in vitro*. Instead, both free viruses and those in complex with antibodies transited across endothelial cells in the BBB model, as observed by confocal microscopy. HIV-1 infectious capacity was considerably altered by the transcytosis process but still detectable, even in the presence of nonneutralizing antibodies. Only virions bound by neutralizing antibodies lacked posttranscytosis infectivity. Overall, our data support the role of neutralizing antibodies in protecting susceptible brain cells from HIV-1 infection despite their inability to inhibit viral BBB endocytic transport.

## Abstract (precis)

HIV-1 can cross the blood-brain barrier (BBB) to penetrate the brain and infect target cells, causing neurocognitive disorders as a result of neuroinflammation and brain damage. The HIV-1 envelope spike gp160 is partially required for viral transcytosis across the BBB endothelium. But do antibodies developing in infected individuals and targeting the HIV-1 gp160 glycoproteins block HIV-1 transcytosis through the BBB? We addressed this issue and discovered that anti-gp160 antibodies do not block HIV-1 transport; instead, free viruses and those in complex with antibodies can transit across BBB endothelial cells. Importantly, we found that only neutralizing antibodies could inhibit posttranscytosis viral infectivity, highlighting their ability to protect susceptible brain cells from HIV-1 infection.

## OBSERVATION

Early postinfection, HIV-1 can invade the central nervous system (CNS) and be transmitted to CNS-resident susceptible cells, such as perivascular macrophages, microglia, astrocytes, and pericytes (1–3). HIV-1 replication in brain target cells may favor establishing a local viral reservoir (4) and causes a neuroinflammation contributing to neurocognitive dysfunctions (1, 5, 6). HIV-1-associated neurocognitive disorders range from asymptomatic and mild neurocognitive impairment to dementia (5). To penetrate into the CNS, HIV-1 must cross the blood-brain barrier (BBB), which is a selective diffusion barrier essential for protecting the CNS from toxic molecules and circulating pathogens as well as for controlling brain homeostasis (7). The BBB is made of capillary endothelial cells joined by tight and adherent junctions, covered by a basal membrane, and surrounded by pericytes and astrocytic endfeet (7). Free and cell-associated HIV-1 virions can translocate across the BBB and enter the CNS via mechanisms common to other neuroinvasive pathogens (8). HIV-1 crosses the BBB following a Trojan horse model in which infected cells transmigrate by diapedesis through intact endothelial cells and by a direct paracellular transversal of the BBB damaged as a result of the infection (9, 10). HIV-1 also traverses the BBB through a transcytosis pathway (1), which may be initiated by viral envelope gp160 glycoproteins binding to proteoglycans (11) and mannose-6-phoshate receptors on endothelial cells (12). Antibodies specific to the HIV-1 envelope spike are rapidly elicited in infected humans, but only those targeting functional gp160 epitopes are neutralizing (13). Moreover, only rare infected individuals develop broadly reactive antibodies neutralizing most HIV-1 strains (13). These broadly neutralizing antibodies (bNAbs) efficiently protect nonhuman primates from infection and decrease viremia in infected humans (14), and thus, they offer promise for HIV-1 prevention and treatment. Whether bNAbs, and more generally anti-gp160 IgG antibodies, interfere with or block HIV-1 transcytosis across the BBB has remained unknown.

## 

### gp160 antibodies do not block HIV-1 transcytosis across the blood-brain barrier.

**FIG 1:**
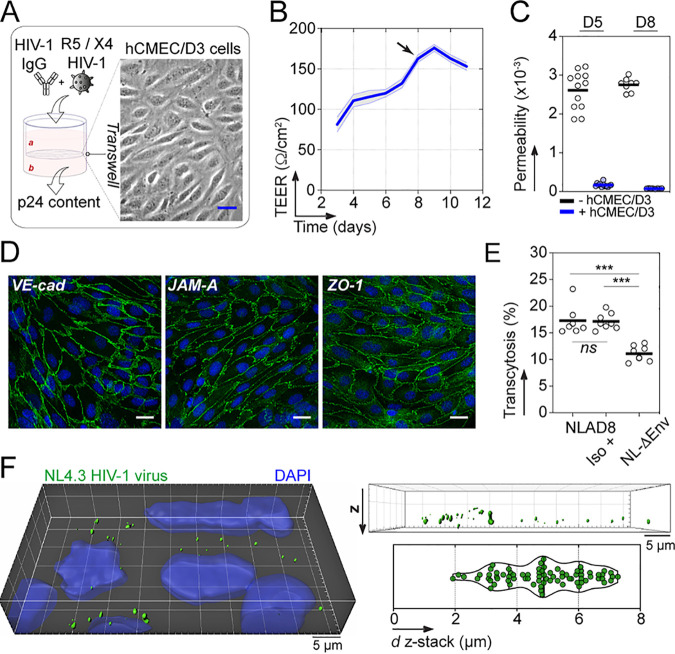
HIV-1 transcytosis across the BBB endothelium. (A) *In vitro* BBB endothelial cell system. The phase-contrast image shows an hCMEC/D3 endothelial cell monolayer separating apical (a) and basal (b) compartments when grown at confluence on a Transwell membrane (bar, 25 μm). HIV-1 virions alone or in the presence of antibodies applied to the apical compartment transcytose and are quantified in the basal medium by p24 ELISA. (B) Transendothelial electrical resistance (TEER) values over time of hCMEC/D3 endothelial cell monolayers. Means of quadruplicate values ± standard errors of the means (SEM) from three independent experiments are shown. Transcytosis experiments were performed at day 8 (black arrow). (C) Dot plots comparing the diffusion of FITC-labeled DEAE-dextran molecules from the apical to the basal compartment when passing through the Transwell membrane without cells (black) and with hCMEC/D3 cells (blue) at day 5 (D5; *n* = 12) and day 8 (D8; *n* = 8). Means of quadruplicate values ± SEM from two or three independent experiments are shown. (D) Immunofluorescence staining of hCMEC/D3 monolayers with DAPI (blue) and anti-VE-cadherin, anti-JAM-A, and anti-ZO-1 antibodies (green) at day 7 of culture. Bar, 20 μm. (E) Dot plots show the percent transcytosis of NLAD8 viruses incubated with or without non-HIV-1 isotype control (Iso) and of NL-ΔEnv alone (8 Transwells each). Means of quadruplicate values from two independent experiments are shown. ***, *P* < 0.001 (Mann-Whitney test); ns, not significant. (F) 3D confocal microscopy image (left) showing nuclei (blue) and NL4.3 HIV-1-GFP viruses (green). Bar, 5 μm. Box (top right) and violin plot (bottom right) show the distribution of fluorescently labeled NL4.3 virions across the z-stack depth of hCMEC/D3 cells using a representative 7.7-μm z-stack confocal microscopy acquisition.

To test the ability of gp160-specific antibodies to block HIV-1 transcytosis across the BBB, we established an *in vitro* model using the human brain microvascular endothelial cell line hCMEC/D3, which reproduces most characteristics of the BBB endothelium (15). hCMEC/D3 cells grown on Transwell culture membranes for a week assembled in confluent and tight monolayers characterized by a low permeability to 40-kDa DEAE-dextran molecules (Fig. 1A to C). hCMEC/D3 cell monolayers formed both tight and adherent junctions, with expression of vascular endothelial (VE)-cadherin, junctional adhesion molecule A (JAM-A), and zonula occludens-1 (ZO-1) (Fig. 1D). HIV-1 transcytosis was assayed at day 8 of culture by exposing cells to CCR5- or CXCR4-tropic HIV-1 virions (NLAD8 and NL4.3 strains, respectively), and measuring the HIV-1 p24 protein content in the basal compartment medium of the Transwell (Fig. 1A). We found that on average, ∼17% of the viral inoculum passing through the porous membrane translocated across the hCMEC/3 endothelium after 4 h (Fig. 1E). In agreement with previous observations (16), envelope-deficient HIV-1 virions (NL-ΔEnv) displayed lower transcytosis capacity (*P* = 0.0003 versus NLAD8) (Fig. 1E), indicating that HIV-1 transcytosis across the BBB partially depends on envelope glycoproteins. Still, HIV-1 can traverse the BBB endothelium through an Env-independent pathway, for which we cannot completely rule out a paracellular transit of a fraction of HIV-1 virions. However, confocal microscopy analyses of green fluorescent protein (GFP)-labeled HIV-1 showed fluorescent virions located in the cytoplasm and scattered across the depth of endothelial cells (Fig. 1F), arguing in favor of viral entry by endocytosis, as previously shown (17). Whether the endothelial cell transcytosis of HIV-1 involves macropinocytosis, interactions with proteoglycans (11), or receptor-mediated endocytosis through mannose-6-phoshate receptors (12), and possibly its coreceptors CCR5 and CXCR4 expressed on hCMEC/3 cells (11, 18), or alternative receptors remains, however, to be precisely determined.

**FIG 2:**
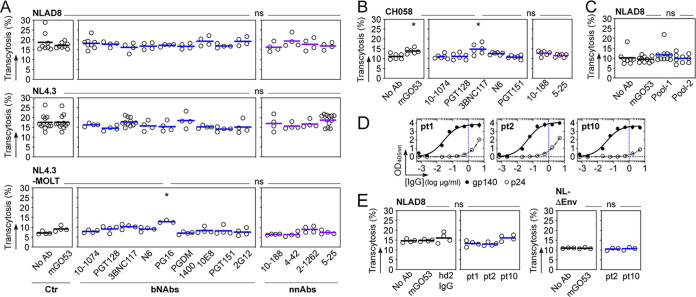
HIV-1 virions in the presence of anti-gp160 antibodies transcytose across the BBB. (A) Dot plots comparing the *in vitro* transcytosis of HIV-1 virions (cell-free NLAD8 [CCR5-tropic]; cell-free and MOLT cell-produced NL4.3 [CXCR4-tropic]) across the hCMEC/D3 cell endothelium (as a percentage of normalized viral input) alone (No Ab) and in the presence of selected bNAbs, NnAbs and non-HIV-1 mGO53 control IgG antibodies. Means of quadruplicate values from one or two experiments are shown. (B) Same as for panel A but with CH058 T/F virions. Means of triplicate values from two experiments are shown. (C) Dot plots comparing the percentage of *in vitro* transcytosis of NLAD8 alone (No Ab) and in the presence of non-HIV-1 isotype control mGO53 and combinations of anti-HIV-1 gp160 antibodies (pool 1, 10-1074, 3BNC117, 10E8 and PGDM1400; pool 2, 10-1074, 3BNC117, 10E8, PGDM1400, 10-188, and 5-25). Means of quadruplicate values from two independent experiments are shown. (D) Graphs showing the ELISA binding of gp140-immunoabsorbed serum IgG antibodies from selected elite neutralizers (pt1, pt2, and pt10) against YU2 gp140 and p24 proteins. Means of duplicate values ± standard deviations (SD) are shown. Vertical dotted lines indicate the IgG concentration (1 μg/ml) used in the experiments presented in panel E. (E) Dot plots comparing the percentage of *in vitro* transcytosis of NLAD8 and NL-ΔEnv virions alone (No Ab) and in the presence of the non-HIV-1 isotype control mGO53, purified serum IgG antibodies from a healthy donor (hd2) (54), and immunopurified polyclonal anti-gp140 antibodies, as shown in panel D. Means of quadruplicate (NLAD8) or triplicate (NL-ΔEnv) values are shown. Groups in panels A, B, C, and E were compared to the no-Ab controls using Dunn's multiple-comparison test. *, 0.035 < *P* < 0.05; ns, not significant.

We next measured HIV-1 transcytosis in the presence of nonneutralizing antibodies (NnAbs) and bNAbs targeting various gp160 epitopes. Unexpectedly, none of the 13 tested single NnAbs and bNAbs showed inhibitory activities against the transport of NLAD8 and NL4.3 virions across hCMEC/D3 cell monolayers (Fig. 2A). The neonatal Fc receptor (FcRn) is expressed on BBB endothelial cells (19), but its role in antibody transcytosis across the BBB is still debated (20). FcRn expression on cultured hCMEC/D3 cells is also greatly reduced compared to that on human primary BBB endothelial cells (21). Thus, as HIV-1 virions alone and bound by antibodies exhibited comparable transcytosis rates, the uptake of antibody-virus complexes by hCMEC/D3 cells is unlikely to occur through the FcRn, as previously shown for mucosal epithelial cell transcytosis (22). Since HIV-1 entry into the CNS is thought to result mainly from HIV-1-infected cells migrating through the BBB (the Trojan horse hypothesis) (1), we then used human T lymphoblastic cells chronically infected with HIV-1 NL4.3 viral strain as a source of virions and tested the same panel of antibodies in the *in vitro* BBB model. As observed with cell-free virus, none of the NnAbs and bNAbs tested as IgG antibodies inhibited the transendothelial transit of cell-associated HIV-1 (Fig. 2A). However, whether HIV-1 gp160 antibodies have the capacity to limit the translocation of HIV-1-infected cells into the brain is a key point that still remains to be resolved. As CNS-invading HIV-1 subpopulations are mainly transmitted/founder (T/F) viruses (23), we measured the transcytosis of CH058 T/F virions and also found the viral endocytic transport across the BBB endothelium to be unaffected by HIV-1 gp160 NnAbs and bNAbs (Fig. 2B). In this regard, it would be interesting to reproduce these experiments with T/F viruses isolated from the cerebrospinal fluid of HIV-1-infected subjects and for which the neutralization sensitivity to some of the bNAbs tested here has been previously measured (24). Finally, we evaluated whether combinations of antibodies recognizing nonoverlapping epitopes would be more effective than single molecules against HIV-1 transcytosis. Neither of the two antibody mixtures composed of 4 bNAbs with and without 2 NnAbs decreased NLAD8 viral transcytosis across hCMEC/D3 endothelial monolayers (Fig. 2C). Similarly, elite neutralizers’ serum IgGs immunopurified against trimeric YU2 gp140 glycoproteins to deplete anti-p24 antibodies blocking p24 HIV-1 detection posttranscytosis had no impact on the intra-endothelial cell migration of NLAD8 virions (Fig. 2D and E).

### HIV-1–bNAb complexes can cross the BBB endothelium but lack infectious potential.

**FIG 3:**
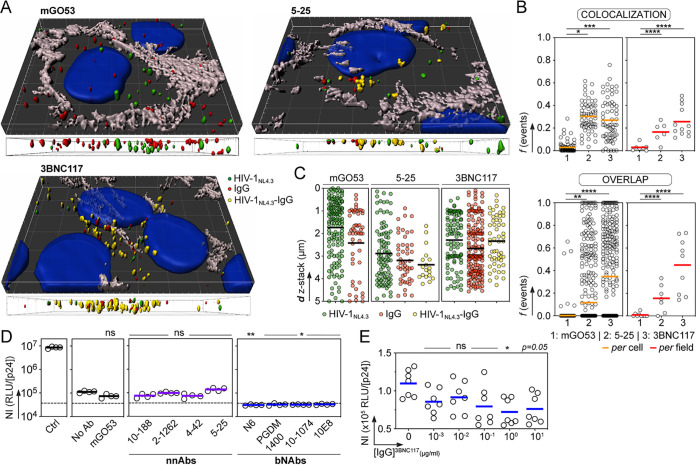
bNAbs neutralize HIV-1 transcytosed through the BBB endothelium. (A) Representative images from the 3D reconstruction of the confocal microscopy experiments on hCMEC/D3 cell monolayers with GFP-labeled HIV-1 (green) in the presence of mGO53, 5-25 and 3BNC117 antibodies (red). Yellow objects represent HIV-1 virions collocating with antibodies. Nuclei and intercellular junctions were stained with DAPI (blue) and anti-VE cadherin antibody (gray), respectively. Side views of the 3D confocal microscopy pictures across the depth of endothelial cells are shown below each image. (B) Graphs comparing colocalization using Pearson coefficient (*r*) and percentage of overlap between fluorescent HIV-1 viruses and mGO53, 5-25, and 3BNC117 antibodies, per cell and per field. Values of the Pearson coefficient and frequency of overlapping virus-IgG objects were compared between groups using Dunn's multiple-comparison and Mann-Whitney tests, respectively. *, *P* < 0.05; **, *P* < 0.01; ***, *P* < 0.001; **** *P* ≤ 0.0001. (C) Dot plots show the number of fluorescent objects corresponding to HIV-1 NL4.3 virus alone (green) or antibodies IgG alone (red) or virus-antibody complexes (HIV_NL4.3_-IgG; yellow) according to the distance across the z-stack depth. Quantification was made using three representative 4- to 5-μm z-stack acquisitions. (D) Dot plots comparing the normalized infectivity (NI; calculated as RLU/p24 concentration [nanograms per milliliter) of HIV-1 virions transcytosed across the BBB in the absence (No Ab) or presence of a non-HIV-1 isotype control (mGO53), HIV-1 gp160 NnAbs, and bNAbs. Ctrl, HIV-1 applied to Transwell membranes without endothelial cells. Each replicate per condition (*n* = 4) was tested in triplicate for infectivity. Dotted lines indicate basal levels of luminescence given by TZM-bl cells alone. (E) Same as for panel D but with various concentrations of 3BNC117 IgG antibodies. Each replicate (*n* = 3 or 4) per independent experiment (*n* = 2) was tested in triplicate for infectivity. Groups in panels D and E were compared to no-Ab controls using Dunn's multiple-comparison test. *, *P* < 0.05; ** *P* < 0.01; ns, non significant.

To investigate the mechanisms of HIV-1 transport in the BBB endothelium in the presence of anti-gp160 antibodies, we performed confocal microscopy experiments using the *in vitro* BBB model with GFP-labeled HIV-1 viruses alone or incubated with the bNAb 3BNC117, the NnAb 5-25, and the non-HIV-1-targeting control IgG mGO53 (Fig. 3A). As expected, viruses and antibodies were observed inside adherent hCMEC/D3 cells and found as antibody-bound HIV-1 virions only with anti-gp160 antibodies regardless of their neutralizing potential (Fig. 2A). Pearson coefficient analyses of the fluorescent objects revealed a significant trend for HIV-1 colocalization with gp160-specific antibodies, predominantly with the bNAb 3BNC117 (26% [*P* < 0.0001] versus 17% [*P* = 0.0045] for 5-25), but not with the isotype control (Fig. 3B). In agreement with this, overlapping virus-antibody fluorescent signals were more frequently detected with 3BNC117 than with 5-25 (45% versus 16% [*P* = 0.0077]) and in less than 1% with the IgG control (Fig. 2B). As for HIV-1 and antibodies alone, virions in complex with 5-25 and 3BNC117 were located in the cytoplasm and distributed across the endothelial cell depth (Fig. 3A and C).

As we observed intracellular fluorescent clusters of HIV-1 with anti-gp160 IgGs by confocal microscopy, we next determined whether HIV-1 transcytosed alone and in the presence of antibodies was still infectious using TZM-bl reporter cells. Infectivity data normalized for virus input by p24 content revealed that the viral transit through hCMEC/D3 endothelial monolayers substantially decreased HIV-1 infectivity compared to virions that only crossed the membrane without hCMEC/D3 cells, with an average drop of 1.2 to 1.4 log_10_ (Fig. 3D). Comparable low but detectable infectivity levels were measured with transcytosed viruses preincubated with anti-gp160 NnAbs (Fig. 3D). However, as opposed to HIV-1 alone and mixed with NnAbs, posttranscytosis infectivity of virions bound by bNAbs reached basal signal levels (Fig. 3D). HIV-1 residual infectivity following BBB intracellular migration was completely inhibited by bNAbs regardless of the targeted neutralizing epitope (Fig. 3D). To estimate the amount of antibodies required to neutralize transcytosed HIV-1, we tested the IgG bNAb 3BNC117 across a broad concentration range. Even if virions were poorly infectious after crossing endothelial cells, we observed a dose-dependent effect of 3BNC117 on viral neutralization posttranscytosis, which was less effective below 1 μg/ml (Fig. 3E).

Collectively, our data show that although the envelope spike of HIV-1 is partially needed for its transcytosis across the BBB, gp160-specific antibodies, singly or in combination, had no measurable effects on the intracellular transport of the virus *in vitro*. HIV-1 virions migrating by endocytosis through the BBB endothelium alone or in the presence of NnAbs remained infectious, but at much lower levels than nontranscytosed viruses. Thus, it is not clear whether transcytosed HIV-1 would support the productive infection of susceptible CNS cells *in vivo*. Alternatively, HIV-1 opsonized by NnAbs could be captured by brain phagocytic cells, such as microglia, and then eliminated by antibody-dependent cellular phagocytosis. Deprived of neutralization capacity, HIV-1-binding antibodies could still promote killing of infected cells crossing the BBB via Fc effector functions. In this regard, HIV-1 NnAbs in the cerebrospinal fluid of infected individuals have been shown to induce the destruction of cells infected by a laboratory-adapted strain by antibody-dependent cellular cytotoxicity (ADCC) (25). However, while lab-adapted strains are commonly sensitive to NnAb-mediated ADCC, primary and T/F viruses are generally resistant (26, 27). On the other hand, we demonstrate in this study that gp160 bNAbs are able to fully neutralize cell-free HIV-1 crossing the BBB. We and others have previously found that the neutralizing activity of anti-gp160 antibodies is essential in protecting target cells from being infected by HIV-1 translocating across mucosal barriers (22, 28, 29). Hence, we propose that the neutralization ability of gp160 antibodies is also a key factor to protect the CNS from HIV-1 infection and spread. Since bNAbs are also potent inducers of Fc-dependent antiviral activities such as ADCC (30, 31), they are the most suitable HIV-1 antibodies for preventing viral brain invasion.

### Antibodies, cells, and viruses.

HIV-1 gp160 bNAbs targeting the CD4bs (3BNC117 [32] and N6 [33]), the *N*-glycan V1/V2 loops (PG16 [34] and PGDM1400 [35]), the *N*-glycan V3 loop (10-1074 [36] and PGT128 [37]), *N*-glycans (2G12 [38]), the gp120/gp41 interface (PGT151 [39]), and the membrane-proximal external region (10E8 [40]), nonneutralizing antibodies targeting the V3 loop crown (10-188 [41], the CD4bs [2-1262], the CD4 induced site [4-42], and the gp41 PID [5-25] [42]), and the non-HIV-1 isotypic control antibody mGO53 (43) were produced as recombinant IgG monoclonal antibodies and purified as previously described (44). Immortalized human cerebral microvascular endothelial cells (hCMEC/D3; Millipore) (18) were cultivated in endothelial basal medium 2 (EBM-2; Lonza) supplemented with 5% heat-inactivated fetal bovine serum (FBS), 1% penicillin-streptomycin (10,000 U/ml), Gibco chemically defined lipid concentrate (1:100), 10 mM HEPES (Thermo Fisher Scientific), 1.4 μM hydrocortisone, 1 ng/ml fibroblast growth factor (basic), and 5 μg/ml l-ascorbic acid (both from Sigma-Aldrich). Culture flasks were pretreated for 1 h with 150 μg/ml of Cultrex rat collagen I (R&D Systems; Bio-Techne) and washed with phosphate-buffered saline (PBS) before addition of cells. hCMEC/D3 cells were detached by trypsinization (0.05% trypsin-EDTA solution; Thermo Fisher Scientific) and cultivated for up to 35 passages (15, 45). TZM-bl cells (no. 8129; NIH AIDS Reagent Program) were cultivated in 10% heat-inactivated FBS–Dulbecco’s modified Eagle medium (DMEM), high glucose, glutaMAX (Thermo Fisher Scientific) supplemented with 1% penicillin-streptomycin (10,000 U/ml; Thermo Fisher Scientific) and routinely passaged by treatment with 0.05% trypsin-EDTA (Thermo Fisher Scientific) or with 0.1% EDTA-PBS prior to the infectivity assay. Chronically NL4.3-infected MOLT cells (46) were grown in RPMI 1640 medium supplemented with 1% penicillin-streptomycin and 10% heat-inactivated FBS (Thermo Fisher Scientific). All aforementioned cell cultures were carried out at 37°C with 5% CO_2_. Infectious molecular clones NLAD8 (no. 11346), NL4.3 (no. 114), and CH058 (no. 11856) (NIH AIDS Reagent Program), viral strain NL-ΔEnv (47, 48), and NL4.3-Gag-eGFP (49) were produced by transfection of 293T cells as previously described (50).

### gp160-specific serum IgG immunopurification.

Trimeric YU2 gp140 protein was produced by transient transfection of Freestyle 293-F suspension cells (Thermo Fisher Scientific) using the polyethylenimine (PEI) precipitation method and were purified by affinity chromatography using high-performance HisPur nickel-nitrilotriacetic acid (Ni-NTA) resin (Thermo Fisher Scientific) as previously described (51). Serum IgG antibodies from HIV-1-positive elite neutralizers (pt1, pt2 [42], and pt10) and were purified by affinity chromatography using protein G Sepharose 4 Fast Flow beads according to the manufacturer’s instructions (GE Healthcare). To conjugate gp140 proteins to Sepharose beads, 2 ml of *N*-hydroxysuccinimide (NHS)-activated Sepharose 4 Fast Flow gel beads (GE Healthcare) was mixed with 200 ml of a 1 mM HCl solution and then washed twice with coupling buffer (0.2 M NaHCO_3_, 0.5 M NaCl [pH 8.3]). NHS-activated beads were incubated 3 h at room temperature on a rolling wheel with 7 mg of purified gp140 in coupling buffer (after 2 successive dialysis cycles). After centrifugation at 3,000 rpm for 3 min, beads were blocked 4 h at 4°C with blocking buffer (0.5 M ethanolamine, 0.5 M NaCl [pH 8.3]) on a rolling wheel. Beads were then washed as follows: twice with low-pH washing buffer (0.1 M sodium acetate, 0.5 M NaCl [pH 5.2]), twice with high-pH washing buffer (0.1 M Tris, 0.5 M NaCl [pH 8.8]), twice with low-pH washing buffer, and twice with PBS. After additional PBS washes, 1 mg of purified IgGs in PBS was incubated with 400 μl of gp140-coupled beads overnight at 4°C on a rolling wheel. Beads were placed in 10-ml chromatography columns (Poly-Prep; Bio-Rad) and washed 10 times with 10 ml of PBS. Antibodies were eluted by addition of 0.5 ml of 0.1 M glycine-HCl (pH 3) and immediately pH neutralized with 1 M Tris base (pH 8) (50 μl per fraction).

High-binding 96-well enzyme-linked immunosorbent assay (ELISA) plates (Costar) were coated overnight with 125 ng/well of purified YU2 gp140 and IIIB p24 proteins (no. 12028; NIH AIDS Reagent Program). After washing with 0.05% Tween-PBS, plates were blocked for 2 h with 2% bovine serum albumin (BSA)–1 μM EDTA–0.05% Tween-PBS (blocking buffer) and then incubated for 2 h with gp140-immunopurified IgG antibodies (250 ng/well in PBS) and seven consecutive 1:3 dilutions in PBS. After washings, plates were revealed by addition of goat horseradish peroxidase (HRP)-conjugated anti-human IgG antibodies (1 μg/ml final in blocking solution; Immunology Jackson ImmunoResearch) and HRP chromogenic substrate (ABTS [2,2′-azinobis(3-ethylbenzthiazoline-6-sulfonic acid)] solution; Euromedex). Experiments were performed with a HydroSpeed microplate washer and a Sunrise microplate absorbance reader (Tecan Männedorf), with optical density measurements made at 405 nm (OD_405_).

### Endothelial cell monolayer formation and monitoring.

Transwell inserts (6.5 mm, 0.4-μm pore polyester membrane; Costar, Corning, Kennebunk, ME) were treated first for 45 min with Cultrex rat collagen I (150 μg/ml in PBS, R&D Systems; Bio-Techne) and then for 45 min with 50 μg/ml fibronectin from bovine plasma in PBS (Sigma-Aldrich) and seeded with 2 × 10^4^ hCMEC/D3 cells. Apical media were changed after 6 days of culture. hCMEC/D3 monolayer formation was routinely monitored by measuring the transendothelial electrical resistance (TEER) at the apical and basolateral poles using an epithelial volt-ohm meter (Millicell ERS-2 system). TEER values of hCMEC/D3-containing inserts, obtained after subtraction of values from inserts alone, were multiplied by 0.3 cm^2^ (surface area of the Transwell) to generate a TEER value in ohms per square centimeter. Paracellular permeability of the hCMEC/D3 endothelium was estimated by measuring the transit of 40-kDa fluorescein isothiocyanate (FITC)-labeled DEAE-dextran (Molecular Probes) as previously described (22, 52). The quantity of DEAE-dextran molecules diffusing into the basolateral compartment was determined using a fluorescence plate reader (Enspire; Perkin Elmer), and permeability coefficients were calculated as previously described (53). The expression of adhesion proteins (tight and adherence junctions) was determined by confocal microscopy. Cells were grown on thick glass slides 12 mm in diameter (0.13 to 0.17 mm; Slabor) for 7 days. Slides were successively fixed for 10 min with 4% and 1% paraformaldehyde for zonula occludens-1 (ZO-1) and vascular endothelial-cadherin (VE-cadherin) staining or for 1 min with methanol-acetone for junctional adhesion molecule A (JAM-A) and then washed with PBS. Following cell permeabilization with 0.05% saponin (Sigma) in PBS–1% bovine serum albumin (BSA)–0.01% (wt/vol) sodium azide buffer, immunostainings were performed using 1:50-diluted anti-VE-cadherin antibody clone F-8, 1:50-diluted anti-JAM-A antibody clone J10.4 (Santa Cruz Biotechnology), 1:50-diluted anti-ZO-1 antibody clone 1A12 (Invitrogen), and 1:100-diluted AF647-conjugated goat anti-mouse IgG antibodies (Thermo Fisher Scientific). Slides were washed three times with PBS between antibody incubations and mounted using DAPI (4′,6-diamidino-2-phenylindole)-Fluoromount-G (Southern Biotech) before observation. Images were acquired with a TCS SP5 fluorescence microscope (Leica Microsystems) with a 63× oil objective with a numerical aperture of 1.4 at the PBI platform (Institut Pasteur).

### HIV-1 endothelial cell transcytosis assay.

hCMEC/D3 cell monolayer transwells with TEER values from 150 to 300 Ω/cm^2^ 8 days postculture were used for the transcytosis assay. HIV-1 viruses (5 ng of p24 HIV-1 cell-free NLAD8, NL4.3, or NL-ΔEnv virions in 160 μl [final volume]) were incubated for 1 h at 37°C with purified recombinant antibodies (66.67 nM final concentration, unless specified otherwise), antibody cocktails, or gp140-immunopurified IgG fractions (6.67 nM final concentration), and mixtures were added to the cell monolayers. In each experiment, HIV-1 virions were also added to inserts with and without hCMEC/D3 cells. After 4 h of incubation at 37°C (or 16 h prior to infectivity testing), media were collected from both upper and lower compartments. Each condition was tested in triplicate or quadruplicate. HIV-1 p24 amount was determined for each transwell with an HIV-1 p24 antigen capture assay (Advanced Bioscience Laboratories, Rockville, MD), using two different dilutions of the basal medium. The percentage of transcytosis as normalized percentage of input was calculated following the formula ([p24]_sample_/[p24]_Ctl_) × 100 (where Ctl is control).

### Confocal microscopy analysis of HIV-1 intra-endothelial cell migration.

hCMEC/D3 cells (1.5 × 10^5^) were cultivated on coverslips (12 mm) for 8 days to achieve optimal confluence. NL4.3-Gag-eGFP viruses (100 ng p24) (49) were incubated for 1 h at 37°C with mGO53 (isotype negative control), 5-25, or 3BNC117 IgGs (66.67 nM final concentration). Mixtures were then applied to the cell monolayers for 4 h at 37°C. After washings with PBS, cells were fixed for 10 min with 4% paraformaldehyde and then with 1% paraformaldehyde before washes in PBS and storage at 4°C. The intracellular immunostaining was performed after permeabilization of the cells with 0.05% saponin (Sigma) in PBS–1% BSA–0.01% (wt/vol) sodium azide buffer. The following antibodies were used for the detection of cellular junctions and bNAbs: VE-cadherin clone F-8 (1:50; Santa Cruz Biotechnology) and AF647-conjugated goat anti-human IgG (1:400; Thermo Fisher Scientific). Secondary antibody was labeled with Cy3-conjugated goat anti-mouse IgG1 (1:400; Jackson Immunoresearch). Between steps, coverslips were washed three times with PBS and mounted using DAPI–Fluoromount-G (Southern Biotech) before observation. z-stack confocal acquisitions were performed on a Leica TCS SP5 microscope with a HCXPLAPO 63× oil objective, NA 1.4, at the UTechS PBI platform (Imagopole, Institut Pasteur). Channel alignments were performed with 0.1-mm tetraSpeck microspheres (blue, green, orange, and dark red; Thermo Fisher Scientific) to control and correct for the chromatic shift. Data were then analyzed after deconvolution using the object analyzer advanced modules of the Huygens Professional software (v14-10; SVI) at the PBI platform (C2RT, Institut Pasteur). For three-dimensional (3D) reconstitutions, Imaris 64 software (v 9.2.0; Bitplane) was used at the Image Analysis Hub (Institut Pasteur).

### *In vitro* HIV-1 infectivity assay.

Infectivity of transcytosed HIV-1 virions in the presence or absence of anti-gp160 or isotypic control IgG antibodies was measured using the TZM-bl cell assay. TZM-bl reporter cells (1 × 10^4^ per well) in full DMEM containing 8 μg/ml of DEAE-dextran were incubated with 200 μl of transcytosed viruses recovered from the basal medium for 48 h at 37°C. In each experiment, wells from transcytosis experiments were tested in triplicate and in parallel with 12.5 mM nevirapine and NLAD8 virus alone (0.05 ng) as controls and incubated with 20 μg/ml of 10-1074 IgG1. After 48 h, cells were lysed, and the assay was developed with the Bright-Glo luciferase assay reagent (Promega). Luminescence signal was measured as relative light units (RLU) using the Enspire microplate luminometer (Perkin Elmer). Normalized infectivity per well was calculated by dividing the mean number of RLU by the p24 concentration (in nanograms per milliliter).

### Statistics.

Percentages of viral transcytosis and posttranscytosis infectivity levels were compared between antibody groups to the “no antibody” control group using Dunn's multiple-comparison test. Percentages of colocalization and overlapping fluorescent events measured by confocal microscopy were compared across groups of antibodies using the Kruskal-Wallis test and the *post hoc* Dunn's multiple-comparison test and using the Mann-Whitney test, respectively. Statistical analyses were performed using GraphPad Prism software (v8.1.2, GraphPad Prism Inc.).
